# Supplementary material for: Systematic characterization of the HOXA9 downstream targets in MLL-r leukemia by noncoding CRISPR screens
Source: Nat Commun. 2023 Nov 28;14:7464. doi: 10.1038/s41467-023-43264-5 (PMC10684515; doi:10.1038/s41467-023-43264-5)
Supplement: Supplementary file 1 — Supplementary Information [file 41467_2023_43264_MOESM1_ESM.pdf]

Systematic characterization of the HOXA9 downstream targets in MLL-r leukemia by noncoding CRISPR screens

Figure Legends for Supplementary Figures.

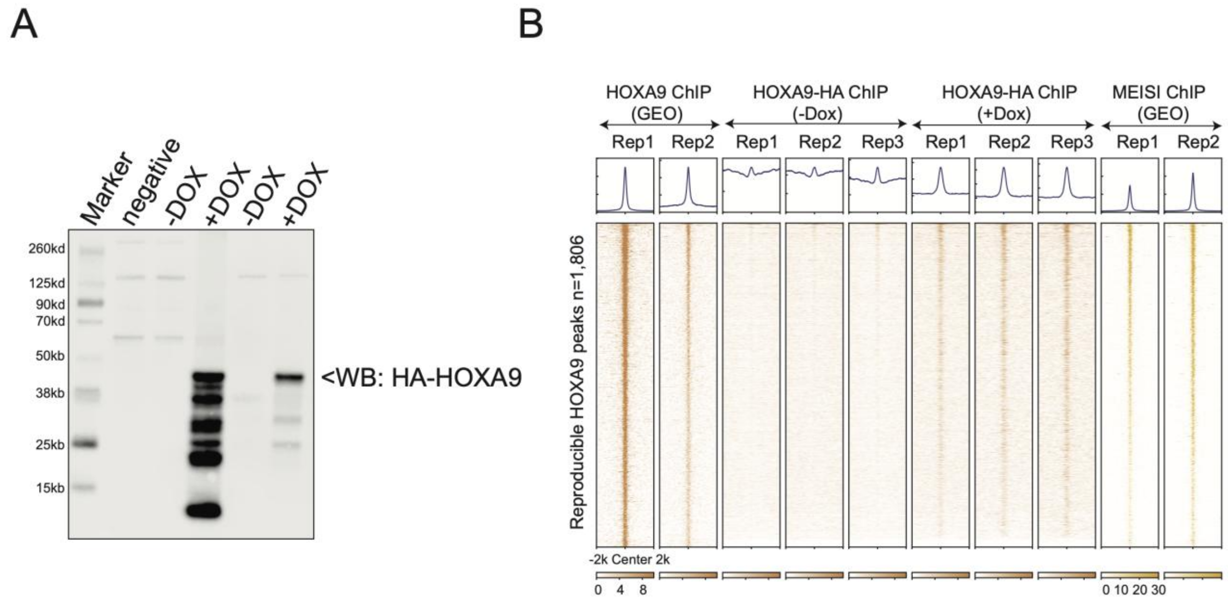

Supplementary Figure 1. Characterization of inducible overexpression system of HOXA9-HA fusion and ChIP-seq. (**A**) Lenti-TRE-3G-TetOn-HOXA9-HA virus was transduced into 293T (lanes 2 and 3) and SEM cells (lanes 4 and 5) followed with 1μg/ml doxycycline treatment for 48 hours. Cell lysates were collected for immunoblotting using antibody against HA. Immunoblotting was done for two times and results are consistent. One representative experiment was shown. (**B**) Heat maps of HOXA9-HA ChIP-seq peaks compared with 1,806 peaks called from publicly available HOXA9 and MEIS1 ChIP-seq. HOXA9 and MEISI ChIP-seq data were from publicly available dataset (GEO: GSE38339).

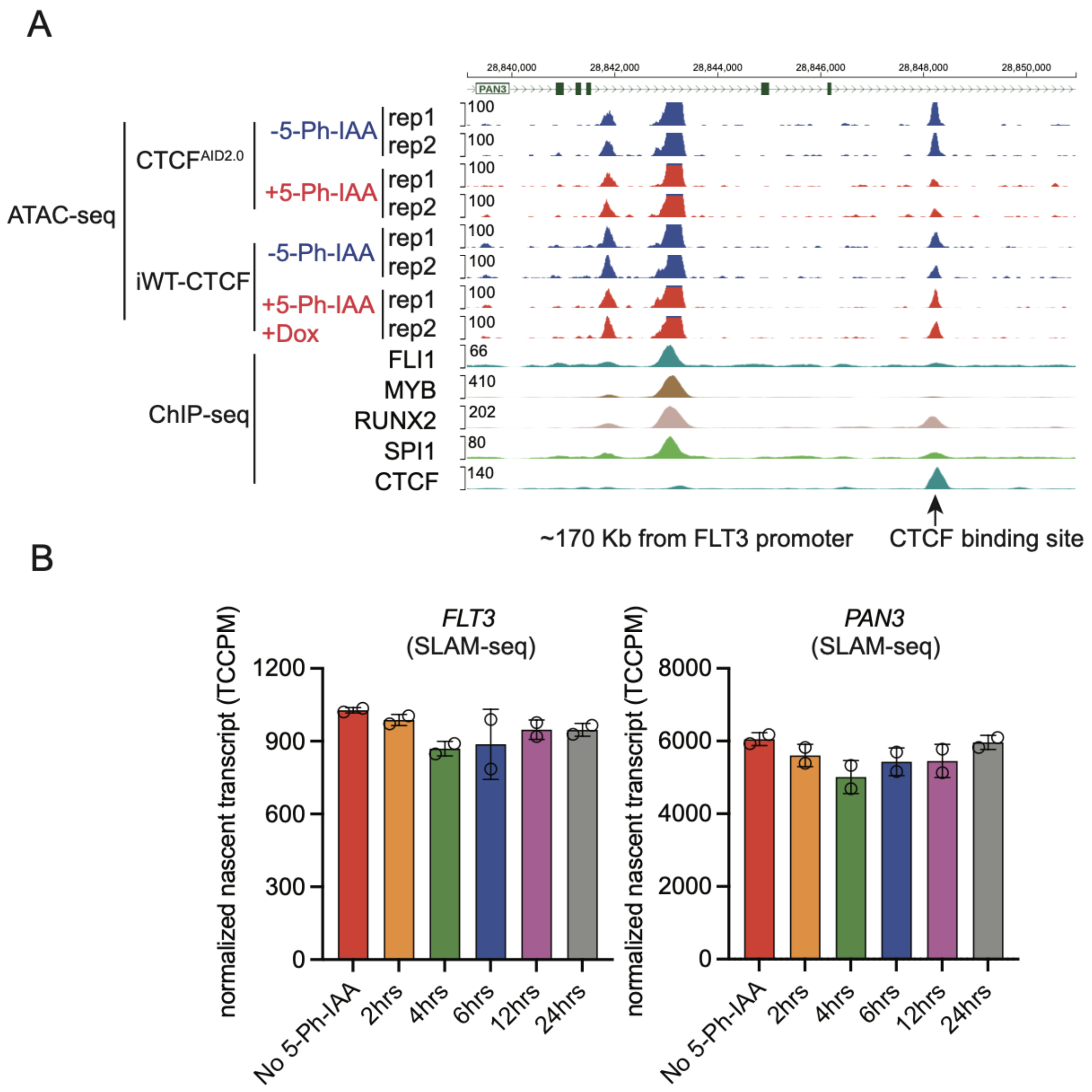

Supplementary Figure 2. CTCF binding in the h-FLT3 region is dispensable for *FLT3* transcription. (**A**) Depletion of CTCF specifically decreased the chromatin accessibility at the target site of the h-FLT3 enhancer, as indicated by ATAC-seq data. ChIP-seq tracks of CTCF, FLI1, MYB, RUNX1 and SPI1 were used to suggest the TF profiling for this locus. (**B**) Depletion of CTCF did not influence the transcription of *FLT3* or *PAN3*, as indicated by SLAM-seq. Nascent transcripts of *FLT3* and *PAN3* were shown in comparison with control cells without auxin (5-Ph-IAA) treatment. Data are shown as two biological replicates.

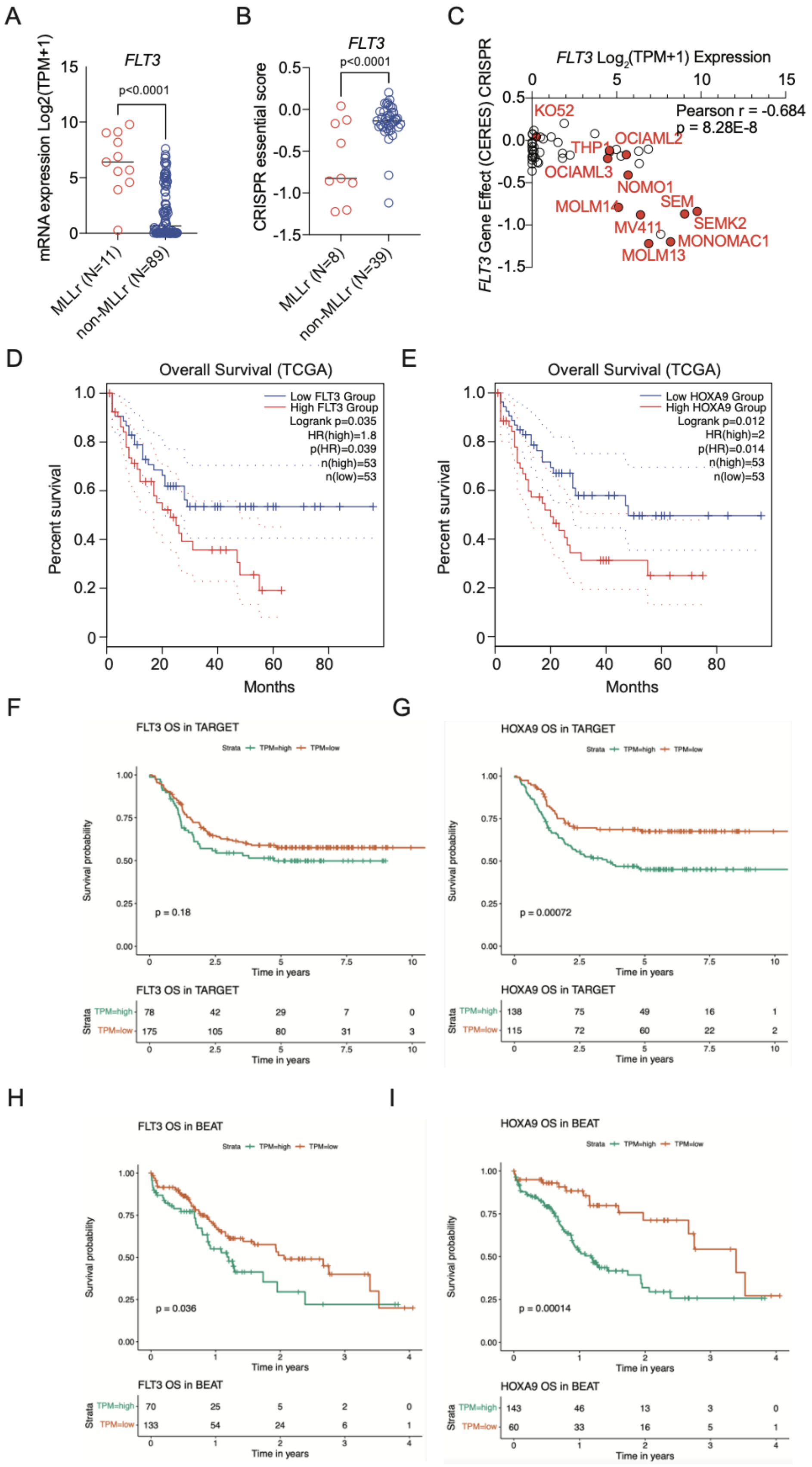
A
FLT3
mRNA expression Log2(TPM+1)
p<0.0001
MLLr (N=11)
non-MLLr (N=89)
B
FLT3
CRISPR essential score
p<0.0001
MLLr (N=8)
non-MLLr (N=39)
C
FLT3 Log2(TPM+1) Expression
FLT3 Gene Effect (CERES) CRISPR
Pearson r = -0.684
p = 8.28E-8
KO52
THP1
OCIAML2
OCIAML3
NOMO1
MOLM14
SEM
MV411
SEMK2
MONOMAC1
MOLM13
D
Overall Survival (TCGA)
Low FLT3 Group
High FLT3 Group
Logrank p=0.035
HR(high)=1.8
p(HR)=0.039
n(high)=53
n(low)=53
Percent survival
Months
E
Overall Survival (TCGA)
Low HOXA9 Group
High HOXA9 Group
Logrank p=0.012
HR(high)=2
p(HR)=0.014
n(high)=53
n(low)=53
Percent survival
Months
F
FLT3 OS in TARGET
Strata TPM=high TPM=low
Survival probability
p = 0.18
Time in years
FLT3 OS in TARGET
TPM=high 78 42 29 7 0
TPM=low 175 105 80 31 3
Time in years
G
HOXA9 OS in TARGET
Strata TPM=high TPM=low
Survival probability
p = 0.00072
Time in years
HOXA9 OS in TARGET
TPM=high 138 75 49 16 1
TPM=low 115 72 60 22 2
Time in years
H
FLT3 OS in BEAT
Strata TPM=high TPM=low
Survival probability
p = 0.036
Time in years
FLT3 OS in BEAT
TPM=high 70 25 5 2 0
TPM=low 133 54 24 6 1
Time in years
I
HOXA9 OS in BEAT
Strata TPM=high TPM=low
Survival probability
p = 0.00014
Time in years
HOXA9 OS in BEAT
TPM=high 143 46 13 3 0
TPM=low 60 33 16 5 1
Time in years

Supplementary Figure 3. Characterization of *FLT3* expression and survival dependency in MLL-r leukemia cells. (**A**) Summary of *FLT3* expression in MLL-r and non-MLL-r human leukemia cell lines in DEPMAP. P values were estimated using a two-tail un-paired *t*-test. Sample size: MLL-r, N=11; non-MLL-r, N=89. Gene expression data were collected from DepMap leukemia cell lines. (**B**) Summary of CRISPR essential scores of *FLT3* in MLL-r and non-MLL-r human leukemia cell lines. Sample size: MLL-r, N=8; non-MLL-r, N=39. CRISPR essential score data were collected from DepMap leukemia cell lines. (**C**) A negative correlation between *FLT3* mRNA expression (x-axis) and survival essential score (y-axis) was confirmed by DepMap data collected from leukemia cell lines. The red dots indicated MLL-r subtypes. MLL-r, N=11. Gene expression and CRISPR essential score data were collected from DepMap leukemia cell lines. (**D-E**) The overall survival of $FLT3^{high}$ and $FLT3^{low}$ patients, $HOXA9^{high}$ and $HOXA9^{low}$ in the TCGA AML patient cohort. $FLT3^{high}$ (N=53) and $FLT3^{low}$ (N=53) for **D**, and $HOXA9^{high}$ (N=53) and $HOXA9^{low}$ (N=53) for **E**. (**F-G**) The overall survival of $FLT3^{high}$ and $FLT3^{low}$, $HOXA9^{high}$ and $HOXA9^{low}$ in the TARGET patient cohort. $FLT3^{high}$ (N=78) and $FLT3^{low}$ (N=175) for **F**, and $HOXA9^{high}$ (N=138) and $HOXA9^{low}$ (N=115) for **G**. (**H-I**) The overall survival of $FLT3^{high}$ and $FLT3^{low}$, $HOXA9^{high}$ and $HOXA9^{low}$ in the BEAT patient cohort. $FLT3^{high}$ (N=70) and $FLT3^{low}$ (N=133) for **H**, and $HOXA9^{high}$ (N=143) and $HOXA9^{low}$ (N=60) for **I**. Comparison of two survival curves was done using a statistical hypothesis test called the log rank test.

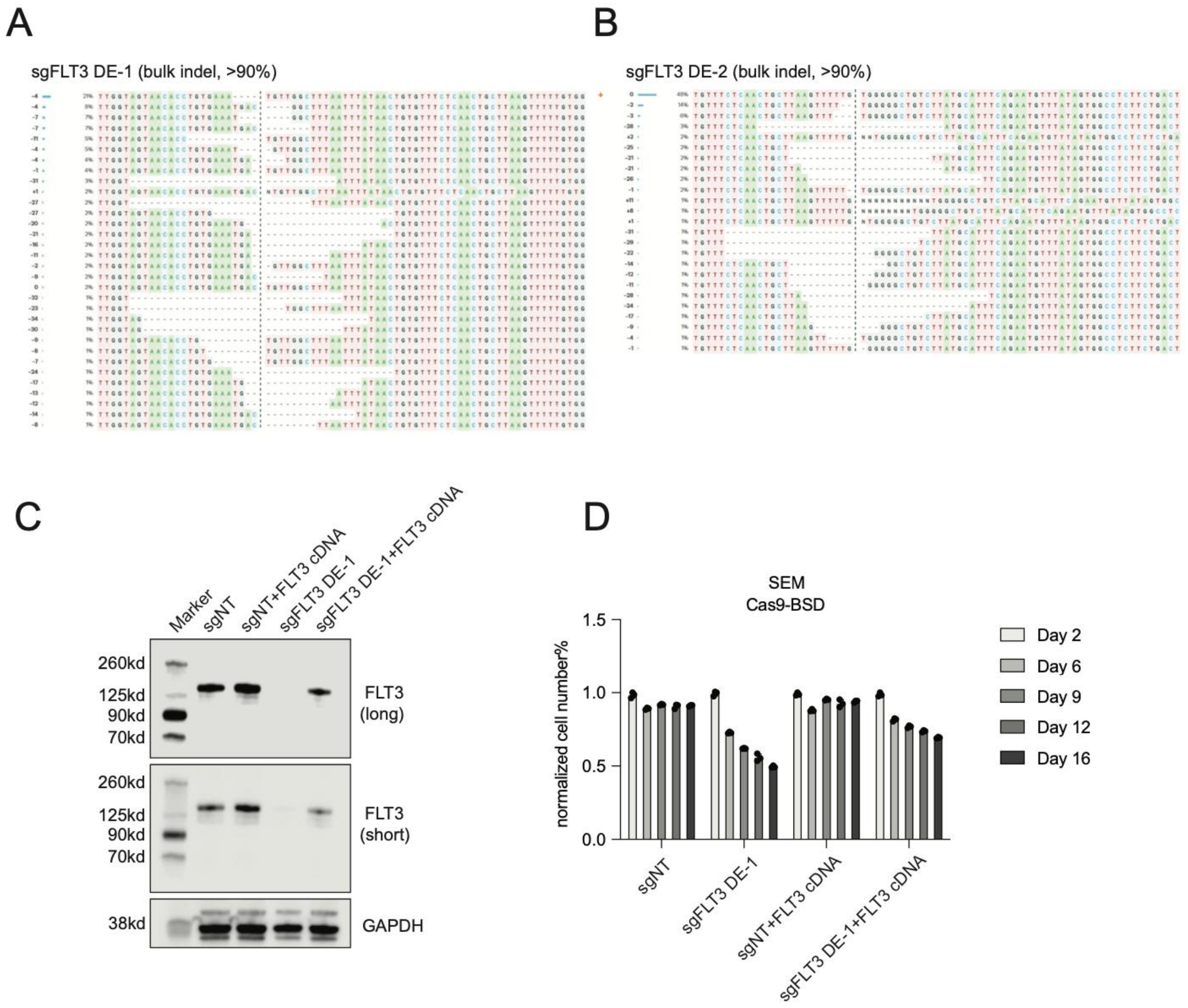

Supplementary Figure 4. Ectopic expression of FLT3 rescued blunted cell growth. (**A-B**) Cas9-expessing cells were transduced with Lenti-sgRNA-CFP-puro and selected for puromycin for three days. Cell pellets were collected for genomic DNA extraction and indel PCR followed by TIDE-seq analysis. (**C**) Ectopic expression re-introduced the expression of *FLT3* cDNA in SEM cells, in which the endogenous expression of *FLT3* had been depleted by CRISPR/Cas9. (**D**) Ectopic expression of *FLT3* cDNA rescued blunted cell growth of SEM cells due to a CRISPR/Cas9-mediated decrease in *FLT3* expression. Percentage of cell numbers was normalized to CFP^+ control cells infected with non-target sgRNA (sgNT). Immunoblotting was done for two times and results are consistent. One representative experiment was shown.

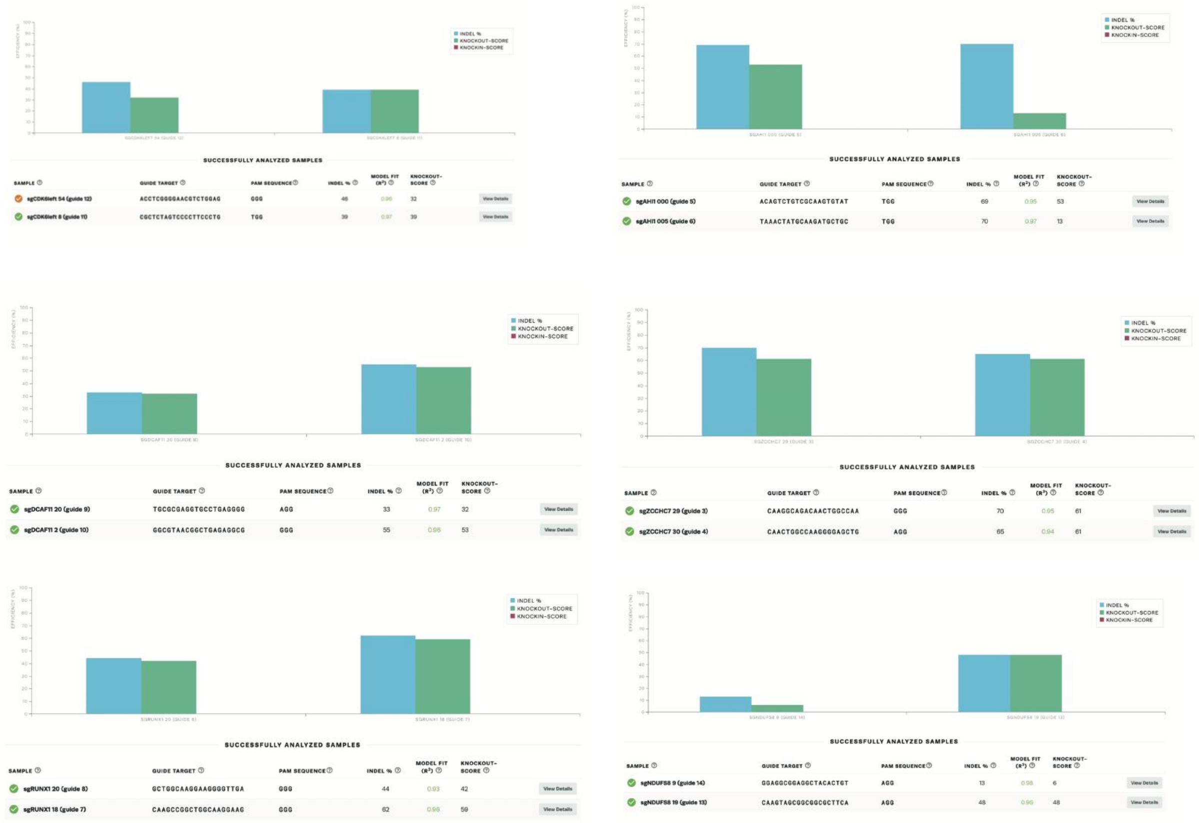

Supplementary Figure 5. Indel validation of CRISPR/Cas9 editing against HOXA9-bound sites. The Cas9-expressing SEM cells were transduced with Lenti-sgRNA-CFP-puro and selected for puromycin for nine days. Cell pellets were collected for genomic DNA extraction and indel PCR followed by TIDE-seq analysis. The sgRNA was designed to target HOXA9-bound sites in the genes *CDK6*, *DCAF11*, *RUNX1*, *AHI1*, *ZCCHC7*, and *NDUFS8*. Indel detection has between confirm twice for Sanger sequencing. The indel frequency was calculated by ICE (Inference of CRISPR Edits), synthego. The P value was calculated based on linear regression of mutation frequency. Each indel analysis was done twice and the result is similar. A representative indel frequency was shown in this figure.

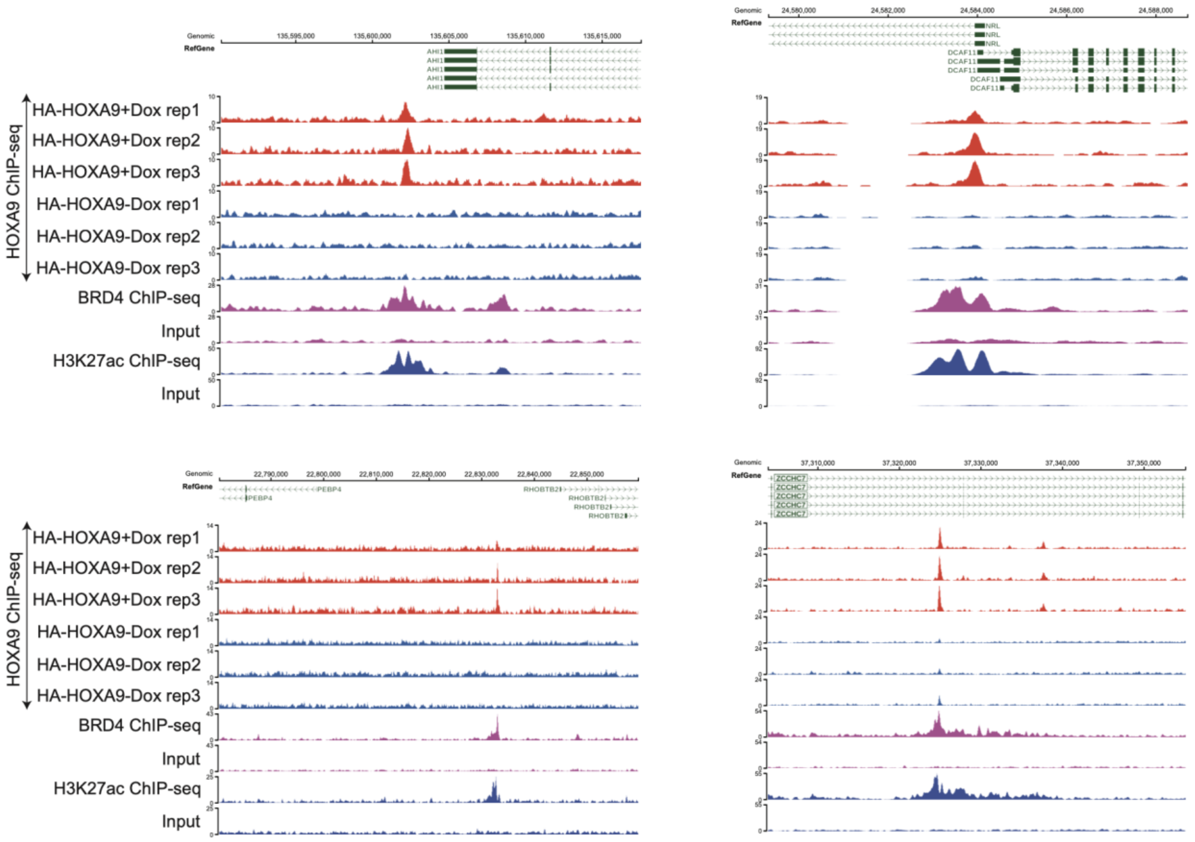

Supplementary Figure 6. Characterizing HOXA9-bound peaks and their enhancer activity. Co-localization of ChIP-seq tracks of HOXA9 and enhancer markers H3K27ac and BRD4 was shown in the genomic loci of *AHI1*, *DCAF11*, *PEBP4,* and *ZCCHC7*. H3K27ac and BRD4 ChIP-seq data were from publicly available dataset (GEO: GSE117864).

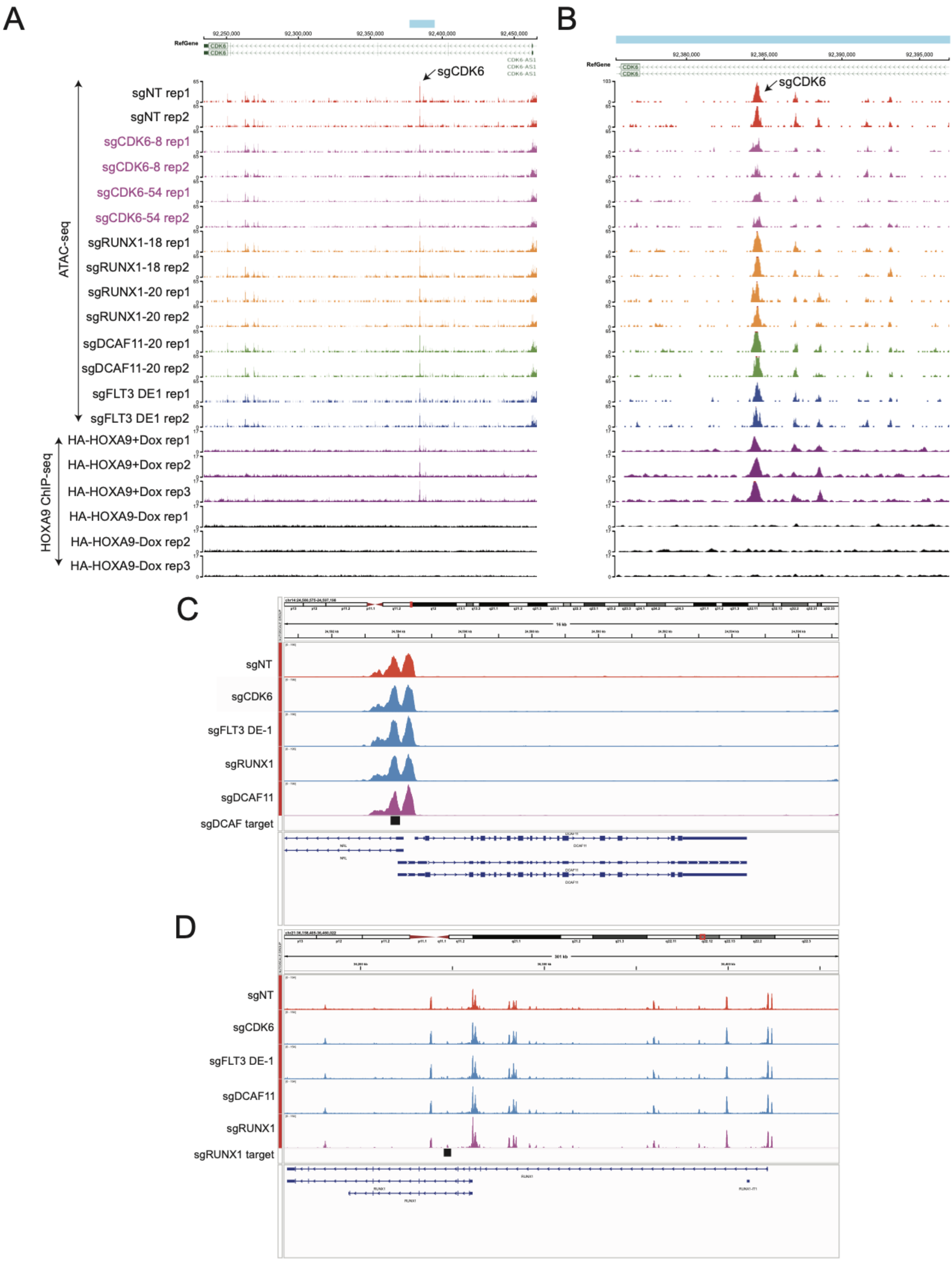

A
B
C
D
RefGene
92,250,000
92,300,000
92,350,000
92,400,000
92,450,000
CDK6
CDK6-AS1
sgCDK6
ATAC-seq
HOXA9 ChIP-seq
sgNT rep1
sgNT rep2
sgCDK6-8 rep1
sgCDK6-8 rep2
sgCDK6-54 rep1
sgCDK6-54 rep2
sgRUNX1-18 rep1
sgRUNX1-18 rep2
sgRUNX1-20 rep1
sgRUNX1-20 rep2
sgDCAF11-20 rep1
sgDCAF11-20 rep2
sgFLT3 DE1 rep1
sgFLT3 DE1 rep2
HA-HOXA9+Dox rep1
HA-HOXA9+Dox rep2
HA-HOXA9+Dox rep3
HA-HOXA9-Dox rep1
HA-HOXA9-Dox rep2
HA-HOXA9-Dox rep3
92,380,000
92,385,000
92,390,000
92,395,000
sgNT
sgCDK6
sgFLT3 DE-1
sgRUNX1
sgDCAF11
sgDCAF target
sgRUNX1 target

Supplementary Figure 7. Chromatin accessibility analysis of CRISPRi-targeted noncoding genome. (**A-B**) The dCas9-KRAB-expressing SEM cells were transduced with Lenti-sgRNA-CFP-puro and selected for puromycin for three days. Cell pellets were collected for ATAC-seq library construction followed by deep sequencing. The sgCDK6 targeting HOXA9-bound site in the intron of CDK6 specifically decreased chromatin accessibility. (**C-D**) CRISPRi-mediated targeting of HOXA9-bound sites in *DCAF11* and *RUNX1* genes did not affect chromatin accessibility.

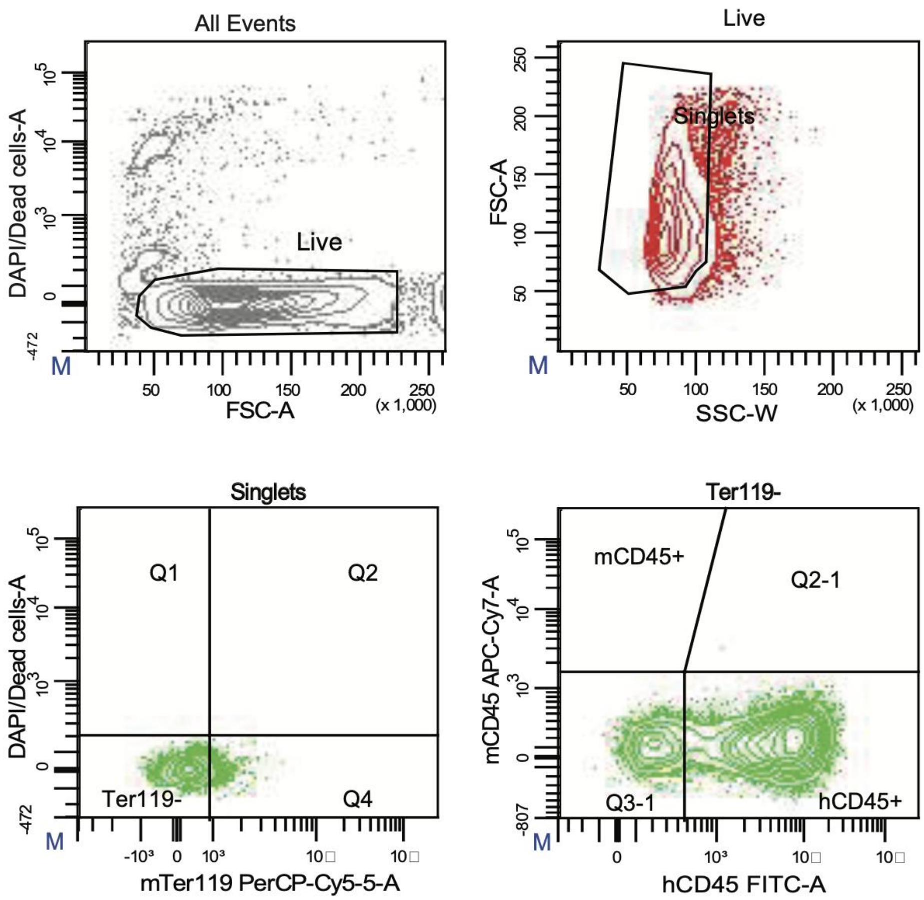

Supplementary Figure 8. Gating strategy for determining percentage of SEM cells in mouse peripheral blood. Mouse retro-orbital blood was RBC lysed and cells were then analyzed by flow cytometry. FSC-A/DAPI was used to determine live cells, and SSC-W/FSC-A was used to determine singlets. DAPI and TER119 were used to determine singlet live lymphocytes (non-red blood cells). Then hCD45 and mCD45 were used to determine percentage of SEM cells.

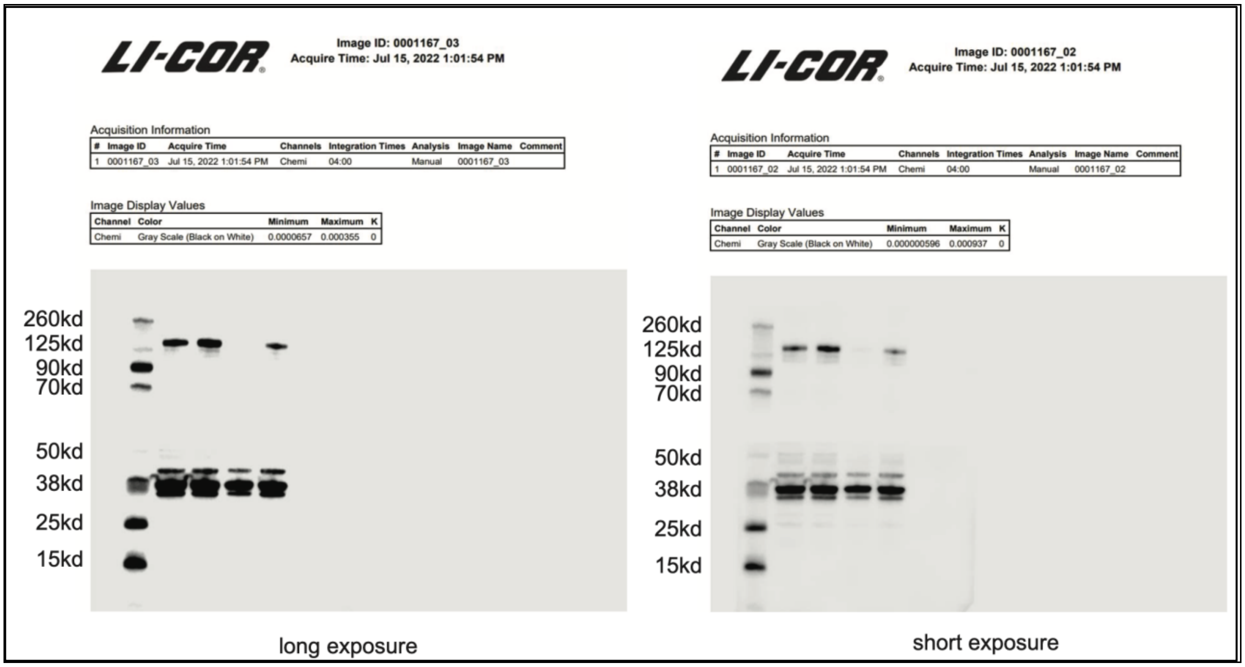

Raw data of uncropped immunoblotting image (refers to Supplementary Figure 4C).
