# Supplementary material for: Systematic characterization of the HOXA9 downstream targets in MLL-r leukemia by noncoding CRISPR screens
Source: Nat Commun. 2023 Nov 28;14:7464. doi: 10.1038/s41467-023-43264-5 (PMC10684515; doi:10.1038/s41467-023-43264-5)
Supplement: Supplementary file 3 — Description of Additional Supplementary Files [file 41467_2023_43264_MOESM3_ESM.pdf]

Figure Legends for Supplementary Data.

Supplementary Data 1. Oligo information related to this study. Oligo information includes cloning primers, Q-PCR primers, sgRNA oligos, and Capture-C oligos.

Supplementary Data 2. CRISPR dropout screen summary. The dCas9-KRAB and Cas9-expressing SEM and MOLM13 cells were infected with the sgRNA library against all 229 HOXA9-bound peaks, followed by 7- and 14-day cultures *in vitro*. The dropout effect was compared between day 7 vs day 0, and day 14 vs day 0 in each cell line by MAGeCK algorithm. Statistical significance was calculated by "MAGeCK test" command. Both sgRNA-based results and gene-based results were provided. For sgRNA-based results, both one-sided and two-sided p-value from null negative binomial distribution were provided. FDR adjusted p-value were computed using Benjamini-Hochberg procedure.

Supplementary Data 3. RNA-seq profiling of SEM cells targeted by sgRNA against HOXA9-bound site in the intronic region of CDK6. The dCas9-KRAB-expressing SEM cells were infected with sgNT and sgCDK6 that target the HOXA9-bound site in the CDK6 intron. RNA samples were collected for RNA-seq analysis and profiled for genome-wide transcriptome analysis. Differentially expressed genes were annotated based on fold change and FDR. The *CDK6* expression is the top reduced gene as expected. The p-value was calculated using raw counts, following by Trimmed Mean of the M-values normalization then limma empirical Bayes analysis pipeline with modified mean-variance trend modeling (using the "voom", "lmFit" and "eBayes" functions from the limma R package). The FDR adjusted p-values were computed using Benjamini-Hochberg procedure.

Supplementary Data 4. Summary of the 229 HOXA9-bound ChIP-seq peaks identified in SEM cells. Reproducible peaks identified from HA-ChIP-seq of three replicates of Doxycycline-induced SEM cells were shown. Detailed information, including genomic coordinates of the closest gene was provided.

Supplementary Data 5. Chromatin accessibility analysis of CRISPRi-targeted noncoding genome. The dCas9-KRAB-expressing SEM cells were transduced with Lenti-sgRNA-CFP-puro and selected for puromycin for three days. Replicate samples were collected from the sgNT, CRISPRi-mediated targeting of HOXA9-bound sites in *DCAF11*, *RUNX1, CDK6, and FLT3* genes. Cell pellets were collected for ATAC-seq library construction, followed by deep

sequencing. Differential ATAC-seq peaks were compared to sgNT groups. Based on peak fold change, P-value, and FDR (q-value column), each differential peak was annotated as described in detail in the method part. The p-value was calculated using raw counts, following by Trimmed Mean of the M-values normalization then limma empirical Bayes analysis pipeline with modified mean-variance trend modeling (using the “voom”, “lmFit” and “eBayes” functions from the limma R package). The FDR adjusted p-values were computed using Benjamini-Hochberg procedure. Details could be found in the original publication (cite https://genomebiology.biomedcentral.com/articles/10.1186/gb-2014-15-2-r29#article-info).

Supplementary Data 6. Summary of publicly available datasets used in this study.
