# Supplementary material for: Systematic characterization of the HOXA9 downstream targets in MLL-r leukemia by noncoding CRISPR screens
Source: Nat Commun. 2023 Nov 28;14:7464. doi: 10.1038/s41467-023-43264-5 (PMC10684515; doi:10.1038/s41467-023-43264-5)
Supplement: Supplementary file 10 — Reporting Summary [file 41467_2023_43264_MOESM10_ESM.pdf]

nature portfolio

Corresponding author(s): Chunliang Li

Last updated by author(s): Aug 1, 2023

Reporting Summary

Nature Portfolio wishes to improve the reproducibility of the work that we publish. This form provides structure for consistency and transparency in reporting. For further information on Nature Portfolio policies, see our Editorial Policies and the Editorial Policy Checklist.

Statistics

For all statistical analyses, confirm that the following items are present in the figure legend, table legend, main text, or Methods section.

n/a	Confirmed	
☐	☒	The exact sample size (*n*) for each experimental group/condition, given as a discrete number and unit of measurement
☐	☒	A statement on whether measurements were taken from distinct samples or whether the same sample was measured repeatedly
☐	☒	The statistical test(s) used AND whether they are one- or two-sided<br>*Only common tests should be described solely by name; describe more complex techniques in the Methods section.*
☐	☒	A description of all covariates tested
☒	☐	A description of any assumptions or corrections, such as tests of normality and adjustment for multiple comparisons
☐	☒	A full description of the statistical parameters including central tendency (e.g. means) or other basic estimates (e.g. regression coefficient) AND variation (e.g. standard deviation) or associated estimates of uncertainty (e.g. confidence intervals)
☐	☒	For null hypothesis testing, the test statistic (e.g. *F*, *t*, *r*) with confidence intervals, effect sizes, degrees of freedom and *P* value noted<br>*Give P values as exact values whenever suitable.*
☒	☐	For Bayesian analysis, information on the choice of priors and Markov chain Monte Carlo settings
☒	☐	For hierarchical and complex designs, identification of the appropriate level for tests and full reporting of outcomes
☒	☐	Estimates of effect sizes (e.g. Cohen's *d*, Pearson's *r*), indicating how they were calculated

Our web collection on statistics for biologists contains articles on many of the points above.

Software and code

Policy information about availability of computer code

Data collection	Flow cytometry (FACS): BD FACS Diva Software V8.0; FlowJo software V10.5.3/10.6.1;<br>MTT (measurement of MTT absorbance) : BioTek Gen5 software V2.0<br>Trim-Galore tool v0.4.4<br>biobambam2 v2.0.87<br>bwa v0.7.17-r1198<br>samtools v1.2<br>bedtools v2.24.0<br>MACS2 tool v2.1.1.20160309<br>TrimGalore v0.6.3<br>STAR v2.7.9a<br>RSEM v1.3.1<br>Picard v2.6.0-SNAPSHOT<br>SPP v1.1
Data analysis	ATAC-seq<br>ATAC-seq data was analyzed as below: Sequenced reads were processed with Trim-Galore tool v0.4.4 (Krueger F. 2012, https://www.bioinformatics.babraham.ac.uk/projects/trim_galore/). Potential adapters were removed, and the 3' ends of reads were quality trimmed with cutadapt (DOI:10.14806/ej.17.1.200) using the quality cutoff of Q20. The first 15 bp of each read were clipped to avoid GC bias and reads

containing more than 50% of soft-clipped bases were removed. Reads were then mapped to the human reference genome (hg19) with bwa v0.7.17-r1198 (PMID: 19451168). Duplicated reads identified with the bamsormadup tool from biobambam2 (v2.0.87, DOI: 10.1186/1751-0473-9-13). Properly paired and uniquely mapped reads were extracted in BAM format with samtools v1.2 (PMID: 19505943). Then, using bedtools v2.24.0 (PMID: 20110278), nucleosome free fragments (defined as fragments <100 bp), were extracted, and MACS2 tool v2.1.1.20160309 (PMID: 18798982) was used to call peaks in narrow format with --extsize 200 ---nomodel -q 0.05 flags (high confidence peaks). Nucleosome free fragments were also used to generate bigWig files for visualization purposes. Separately, ATAC-seq peaks were also called using more relaxed criteria, setting the -q flag to 0.5, which are here referred to as low-confidence peaks. Finally, the reproducible highconfidence peaks between the biological replicates from the same condition were identified as those that, for the same genomic region, either had high or low confidence peaks called in all other biological replicates. Reproducible peaks from each condition were then merged into one collection of reproducible peaks.

RNA-seq
Paired-end 101-cycle sequencing was performed on the NovaSeq 6000 sequencer following the manufacturer's instructions (Illumina). Raw reads were first trimmed using TrimGalore (v0.6.3) with parameters "–paired –retain_unpaired." Filtered reads were then mapped to the Homo sapiens reference genome GRCh37.p13 using STAR (v2.7.9a). Gene-level read quantification was done using RSEM (v1.3.1) on the Gencode annotation v19. To identify the differentially expressed genes, normalization factors were first estimated using the TMM and genes with CPM≤1 in all samples were removed. Next, the TMM normalization factors and raw counts were then used for the Limma-voom analysis using the "voom," "lmFit," and "eBayes" functions from the limma R package. Gene set enrichment analysis (GSEA) was performed using the MsigDB database (v7.1). Differentially expressed genes were ranked based on their log2(FC). The principal component analysis (PCA) plots were generated from the TMM normalized data. Based on the log2(CPM) data, we ranked the genes based on their median absolute deviation (using the "mad" function in R) as it is a more robust statistic against outliers. The log2(CPM) of the top 3000 variable genes was passed to the "prcomp" function to do PCA analysis. The first two principal components were used to generate the PCA plots.

ChIP-seq
ChIP-seq data was analyzed as below: Single-end reads of 100bp were mapped human genome hg19(GRCh37-lite) by BWA (version 0.7.12-r1039, default parameter), duplicated reads were then marked with Picard(version 2.6.0-SNAPSHOT) and only non-duplicated reads have been kept by samtools (parameter "-q 1 -F 1024" version 1.2). We followed ENCODE guideline for quality control and confirmed high quality (>50 million uniquely mapped reads each sample).We extend reads to fragment size detected by SPP(v1.1) and generate bigwig files normalized to 15 million reads.

Hi-C
Raw sequence data were mapped and processed using Juicer v1.5 with default parameters. The Hi-C data and MboI cut sites were mapped to hg19. Replicates data were first processed separately. After confirmation of good reproducibility by HiC-Spector, we merged the replicates and re-processed as combined results.

Capture C: Paired-end reads of 150bp were first trimmed for adapters by cutadapt(version 1.9, paired-end mode, default parameter with " -m 6 -O 25 "). Then trimmed seqeunces were processed by HiC-Pro using human genome hg19 with restriction fragments annotation based on MboI(^GATC).We then generate bigwig files using make_viewpoints.py script from HiC-Pro.We used "chr13:28674037-28674157(P5)" or "chr13:28674930-28675050(P6)" or both as viewpoint for Foxp3 promoter. bigwig tracks height was normalized to 100k contact within 5 million bp, that is we double the track height if we observed a sample have 50k contact within 5 million bp from viewpoint.

MTT data was analyzed by Graphpad Prism (version 9).

For manuscripts utilizing custom algorithms or software that are central to the research but not yet described in published literature, software must be made available to editors and reviewers. We strongly encourage code deposition in a community repository (e.g. GitHub). See the Nature Portfolio guidelines for submitting code & software for further information.

Data

Policy information about availability of data

All manuscripts must include a data availability statement. This statement should provide the following information, where applicable:

- Accession codes, unique identifiers, or web links for publicly available datasets
- A description of any restrictions on data availability
- For clinical datasets or third party data, please ensure that the statement adheres to our policy

The CRISPR screen, Capture-C, ChIP-seq, ATAC-seq, and RNA-seq data generated in this study have been deposited in NCBI GEO under super series (GSE215928) (https://www.ncbi.nlm.nih.gov/geo/query/acc.cgi?acc=GSE215928). The HiC publicly available data used in this study are available in the GEO (GSE138862)(https://www.ncbi.nlm.nih.gov/geo/query/acc.cgi?acc=GSE138862). ATAC-seq publicly available data used in this study are available in the GEO (GSE74912)(https://www.ncbi.nlm.nih.gov/geo/query/acc.cgi?acc=GSE74912); (GSE129066)(https://www.ncbi.nlm.nih.gov/geo/query/acc.cgi?acc=GSE129066); (GSE153237)(https://www.ncbi.nlm.nih.gov/geo/query/acc.cgi?acc=GSE153237). Transcription factor ChIP-seq publicly available data used in this study are available in the GEO (GSE117864)(https://www.ncbi.nlm.nih.gov/geo/query/acc.cgi?acc=GSE117864). H3K27ac ChIP-seq publicly available data used in this study are available in the GEO (GSE17312)(https://www.ncbi.nlm.nih.gov/geo/query/acc.cgi?acc=GSE17312), (GSE80779)(https://www.ncbi.nlm.nih.gov/geo/query/acc.cgi?acc=GSE80779), (GSE65138)(https://www.ncbi.nlm.nih.gov/geo/query/acc.cgi?acc=GSE65138), (GSE79899)(https://www.ncbi.nlm.nih.gov/geo/query/acc.cgi?acc=GSE79899), (GSE109492)(https://www.ncbi.nlm.nih.gov/geo/query/acc.cgi?acc=GSE109492), (GSE137652)(https://www.ncbi.nlm.nih.gov/geo/query/acc.cgi?acc=GSE137652); (GSE111293)(https://www.ncbi.nlm.nih.gov/geo/query/acc.cgi?acc=GSE111293). The remaining data are available within the Article, Supplementary Information or Source Data file.

Research involving human participants, their data, or biological material

Policy information about studies with human participants or human data. See also policy information about sex, gender (identity/presentation), and sexual orientation and race, ethnicity and racism.

Reporting on sex and gender	N.A

Reporting on race, ethnicity, or other socially relevant groupings	N.A
Population characteristics	N.A
Recruitment	N.A
Ethics oversight	N.A

Note that full information on the approval of the study protocol must also be provided in the manuscript.

Field-specific reporting

Please select the one below that is the best fit for your research. If you are not sure, read the appropriate sections before making your selection.

☒ Life sciences ☐ Behavioural & social sciences ☐ Ecological, evolutionary & environmental sciences

For a reference copy of the document with all sections, see nature.com/documents/nr-reporting-summary-flat.pdf

Life sciences study design

All studies must disclose on these points even when the disclosure is negative.

Sample size	Sample size was determined on the basis of data and sample availability
Data exclusions	No data was excluded
Replication	All in vitro experiments have been individually repeated at least three times. All attempts of replication were successful. In vivo experiments were repeated twice with similar results.
Randomization	Randomization is not relevant because all cell lines and biological samples were treated in the same manner.
Blinding	Experiments were not blinded in order to allow the investigators to have accurate identification of samples and to ensure the correct data collection.

Reporting for specific materials, systems and methods

We require information from authors about some types of materials, experimental systems and methods used in many studies. Here, indicate whether each material, system or method listed is relevant to your study. If you are not sure if a list item applies to your research, read the appropriate section before selecting a response.

Materials & experimental systems

n/a	Involved in the study	
☐	☒	Antibodies
☐	☒	Eukaryotic cell lines
☒	☐	Palaeontology and archaeology
☐	☒	Animals and other organisms
☒	☐	Clinical data
☒	☐	Dual use research of concern
☒	☐	Plants

Methods

n/a	Involved in the study	
☐	☒	ChIP-seq
☐	☒	Flow cytometry
☒	☐	MRI-based neuroimaging

Antibodies

Antibodies used	mTER119-PerCP-Cy5.5 (BD Pharmingen, #560512, Clone TER-119, dilution ratio: 1:100), mCD45-APC-Cy7 (BD Pharmingen, #557659, Clone 30-F11, dilution ratio: 1:100), CD45-FITC (BD Pharmingen, #555482, Clone HI30, dilution ratio: 1:100), GAPDH (Thermo Fisher Scientific #AM4300, 1:5,000), AID (MBL #M214-3, 1:2,000), or FLT3 (Cell Signaling, #3462, 1:1,000), H3K27Ac (ActiveMotif, #39133, 1:1,000), Secondary horseradish peroxidase (HRP)-conjugated sheep anti-mouse IgG (GE Healthcare, #NA931V, 1:5000), Secondary horseradish peroxidase (HRP)-conjugated donkey anti-Rabbit IgG (GE Healthcare, #NA934V, 1:5000).
Validation	CD45-FITC (BD Pharmingen, #555482, Clone HI30) was validated in human cells by Flow cytometry<br>https://www.bdbiosciences.com/en-au/products/reagents/flow-cytometry-reagents/research-reagents/single-color-antibodies-ruo/fitc-mouse-anti-human-cd45.555482<br>mouse ER119-PerCP-Cy5.5 (BD Pharmingen, #560512, Clone TER-119) was validated in mouse cells by Flow cytometry<br>https://www.bdbiosciences.com/en-ca/products/reagents/flow-cytometry-reagents/research-reagents/single-color-antibodies-ruo/

percp-cy-5-5-rat-anti-mouse-ter-119-erythroid-cells.560512
mCD45-APC-Cy7 (BD Pharmingen, #557659, Clone 30-F11) was validated in mouse cells by Flow cytometry
https://www.bdbiosciences.com/en-us/products/reagents/flow-cytometry-reagents/research-reagents/single-color-antibodies-ruo/apc-cy-7-rat-anti-mouse-cd45.557659
GAPDH (Thermo Fisher Scientific #AM4300) was validated in human cells by immunoblotting.
https://www.thermofisher.com/antibody/product/GAPDH-Antibody-clone-6C5-Monoclonal/AM4300
AID (MBL #M214-3) was validated using Ecoli expressed proteins by immunoblotting.
https://www.mblbio.com/bio/g/dtl/A/?pcd=M214-3
FLT3 (Cell Signaling, #3462) was validated in human SEM cells and mouse Baf3/FLT3 transfected cells by immunoblotting.
https://www.cellsignal.com/products/primary-antibodies/flt3-8f2-rabbit-mab/3462
H3K27Ac (ActiveMotif, #39133) was validated in human cells by immunoblotting.
https://www.activemotif.com/catalog/details/39133/histone-h3-acetyl-lys27-antibody-pab
Secondary horseradish peroxidase (HRP)-conjugated sheep anti-mouse IgG was validated for multiple mouse origin antibodies.
GE Healthcare, Conjugated Antibody, ECL Mouse IgG, HRP-linked whole Ab (from sheep), NA931-1ML
Secondary horseradish peroxidase (HRP)-conjugated donkey anti-Rabbit IgG was validated for multiple rabbit origin antibodies.
https://us.vwr.com/assetsvc/asset/en_US/id/16614249/contents

Eukaryotic cell lines

Policy information about cell lines and Sex and Gender in Research

Cell line source(s)	REH (CRL-8286), Nalm6 (CRL-3273) and MV411 (CRL-9591) cell lines were originally purchased from ATCC. SEM (DSMZ, ACC-546), OCI-AML2 (DSMZ, ACC-99) and 697 (DSMZ, ACC-42) cell lines were originally purchased from DSMZ. MOLM13 (ABC-TC517S) was originally purchased from ACCEGEN. GM12878 was from Coriell Institute.
Authentication	We have done authentication for all the cell lines used in this study by STR analysis.
Mycoplasma contamination	All cell lines are negative for Mycoplasma confirmed by RT-PCR.
Commonly misidentified lines (See ICLAC register)	No commonly misidentified lines were used in this study.

Animals and other research organisms

Policy information about studies involving animals; ARRIVE guidelines recommended for reporting animal research, and Sex and Gender in Research

Laboratory animals	This study used NOD.Cg-Prkdcscid Il2rgtm1Wjl/SzJ (NSG) mice and all were female and 8-12 weeks old. Mice were maintained in SPF grade St. Jude Animal Facility in 12h/12h dark and light cycle.
Wild animals	This study didn't involve any wild animals.
Reporting on sex	All NSG mice used in this study are female. NSG male mice are showing aggressive behavior than female settings particularly implanted with leukemia and developed disease progression. To avoid potential physical fighting and injury followed with infection, only female mice were included in this type of studies as a default setting.
Field-collected samples	This study didn't involve any field-collected samples.
Ethics oversight	The study was conducted under the ethical approval for animal research by the Animal Care & Use Committee (ACUC) of St. Jude Children's Research Hospital.

Note that full information on the approval of the study protocol must also be provided in the manuscript.

Plants

Seed stocks	N.A
Novel plant genotypes	N.A
Authentication	N.A

ChIP-seq

Data deposition

☒ Confirm that both raw and final processed data have been deposited in a public database such as GEO.

☐ Confirm that you have deposited or provided access to graph files (e.g. BED files) for the called peaks.

Data access links *May remain private before publication.*	https://www.ncbi.nlm.nih.gov/geo/query/acc.cgi?acc=GSE216031 (reviewer token: urehycoahfuzzkf).

Files in database submission

GSM7635410 HOXA9-SEM-TRE3G-HoxAID-HA-zeo-minuxDox-Rep1
GSM7635411 HOXA9-SEM-TRE3G-HoxAID-HA-zeo-minuxDox-Rep2
GSM7635412 HOXA9-SEM-TRE3G-HoxAID-HA-zeo-minuxDox-Rep3
GSM7635413 HOXA9-SEM-TRE3G-HoxAID-HA-zeo-plusDox-Rep1
GSM7635414 HOXA9-SEM-TRE3G-HoxAID-HA-zeo-plusDox-Rep2
GSM7635415 HOXA9-SEM-TRE3G-HoxAID-HA-zeo-plusDox-Rep3
GSM7635416 INPUT-SEM-TRE3G-HoxAID-HA-zeo-minuxDox-Rep1
GSM7635417 INPUT-SEM-TRE3G-HoxAID-HA-zeo-minuxDox-Rep2
GSM7635418 INPUT-SEM-TRE3G-HoxAID-HA-zeo-minuxDox-Rep3
GSM7635419 INPUT-SEM-TRE3G-HoxAID-HA-zeo_PlusDox-Rep1
GSM7635420 INPUT-SEM-TRE3G-HoxAID-HA-zeo-plusDox-Rep2
GSM7635421 INPUT-SEM-TRE3G-HoxAID-HA-zeo-plusDox-Rep3
GSM6656478_H3K27Ac-AB1-SJMLL015091_X2-MLL-AF4.bw
GSM6656479_H3K27Ac-AB1-SJMLL066702_C2-SEM.bw
GSM6656480_H3K27Ac-AB1-SJMLL066703_C2-MOLM13.bw
GSM6656481_H3K27Ac-AB1-SJMLL066704_C2-OCI-AML2.bw
GSM6656482_INPUT-SJMLL015091_X2-MLL-AF4.bw
GSM6656483_INPUT-SJMLL066702_C2-SEM.bw
GSM6656484_INPUT-SJMLL066703_C2-MOLM13.bw
GSM6656485_INPUT-SJMLL066704_C2-OCI-AML2.bw

Genome browser session
(e.g. UCSC)

N.A

Methodology

Replicates

N.A

Sequencing depth

Sample Reads Mapped NonDupMapped Mpd% Dup%
INPUT-SEM-TRE3G-HoxAID-HA-zeo-minuxDox_R1 68.4 66.9 59.2 97.80% 11.60%INPUT-SEM-TRE3G-HoxAID-HA-zeo-minuxDox_R2 76.2 74.5 63.8 97.80% 14.40%INPUT-SEM-TRE3G-HoxAID-HA-zeo-minuxDox_R3 78.8 77.1 66.8 97.80% 13.40%INPUT-SEM-TRE3G-HoxAID-HA-zeo_PlusDox_R1 85.1 83.3 72.2 97.90% 13.30%INPUT-SEM-TRE3G-HoxAID-HA-zeo-plusDox_R2 85.6 83.7 72.2 97.80% 13.70%INPUT-SEM-TRE3G-HoxAID-HA-zeo-plusDox_R3 57.8 56.5 49.8 97.80% 11.90%HOXA9-SEM-TRE3G-HoxAID-HA-zeo-minuxDox_R1 78.4 76.8 65.5 98.00% 14.80%HOXA9-SEM-TRE3G-HoxAID-HA-zeo-minuxDox_R2 72.4 70.8 61.8 97.90% 12.80%HOXA9-SEM-TRE3G-HoxAID-HA-zeo-minuxDox_R3 84.7 83 71.1 98.00% 14.30%HOXA9-SEM-TRE3G-HoxAID-HA-zeo-plusDox_R1 61.3 60 52.6 97.90% 12.40%HOXA9-SEM-TRE3G-HoxAID-HA-zeo-plusDox_R2 43 42.1 37 98.00% 12.20%HOXA9-SEM-TRE3G-HoxAID-HA-zeo-plusDox_R3 39.7 38.9 34.7 98.00% 10.80%
H3K27Ac-AB1-SJMLL015091_X2-MLL-AF4 135604305 123970943 60599603 91.42% 51.12%
H3K27Ac-AB1-SJMLL066702_C2-SEM 134375120 125888061 81578817 93.68% 35.20%
H3K27Ac-AB1-SJMLL066703_C2-MOLM13 130050308 121005791 72437959 93.05% 40.14%
H3K27Ac-AB1-SJMLL066704_C2-OCI-AML2 145151586 132214324 86947633 91.09% 34.24%
INPUT-SJMLL015091_X2-MLL-AF4 136969804 127943212 102885478 93.41% 19.59%
INPUT-SJMLL066702_C2-SEM 104464281 98951278 84338378 94.72% 14.77%
INPUT-SJMLL066703_C2-MOLM13 126015671 117681094 100075505 93.39% 14.96%
INPUT-SJMLL066704_C2-OCI-AML2 141533078 132256786 110742246 93.45% 16.27%

Antibodies

HA-antibody magnetic beads (thermofisher 88837);H3K27Ac (ActiveMotif,39133)

Peak calling parameters

Briefly, reads were mapped to the human genome hg19(GRCh37-lite) by BWA (version 0.7.12-r1039, default parameter). Duplicated reads were marked with Picard (version 2.6.0-SNAPSHOT), and only non-duplicated reads were kept by samtools (parameter "-q 1 -F 1024" version 1.2). MACS2 (version 2.1.1.20160309) was used for peak calling. To ensure reproducibility, reproducible peaks for each group were finalized as only peaks retained if called with a stringent cutoff (-q 0.05) in one sample and at least called with a lower cutoff (-q 0.5) in the other samples.

Data quality

We followed ENCODE guideline for quality control and confirmed high quality (>50 million uniquely mapped reads each sample).

Software

BWA (version 0.7.12-r1039, default parameter); Picard (version 2.6.0-SNAPSHOT); MACS2 (version 2.1.1.20160309); samtools (parameter "-q 1 -F 1024" version 1.2)

Flow Cytometry

Plots

Confirm that:

☒ The axis labels state the marker and fluorochrome used (e.g. CD4-FITC).

☒ The axis scales are clearly visible. Include numbers along axes only for bottom left plot of group (a 'group' is an analysis of identical markers).

☒ All plots are contour plots with outliers or pseudocolor plots.

☒ A numerical value for number of cells or percentage (with statistics) is provided.

Methodology

Sample preparation	The description of sample preparation can be found in the methods section.<br>For flow analyses of cell lines from xenograft mouse model, cells were resuspended in FACS buffer (PBS supplemented with 2% Fetal Calf Serum and 0.25mM EDTA), they were then stained with proper antibodies and washed twice with FACS buffer before being subjected to flow analyses.
Instrument	BD FACS Aria IIIu machine.
Software	BD FACS Diva Software and FlowJo.
Cell population abundance	Only populations with sufficient number of cells (event) were analyzed post-sorting.
Gating strategy	To determine human blast% in peripheral blood, 30-50 uL mouse peripheral blood was collected. after RBC lysis, FSC-A/SSC-A was used to determine lymphocytes, after gating for live lymphocytes (DAPI/mTER119 double negative), SSC-W/FSC-A was used to determine singlets, then hCD45/mCD45 was used to determine human blast%, mCD45 was used to confirm.

☒ Tick this box to confirm that a figure exemplifying the gating strategy is provided in the Supplementary Information.
